# Distribution and diversity of mosquitoes and Oropouche-like virus infection rates in an Amazonian rural settlement

**DOI:** 10.1371/journal.pone.0246932

**Published:** 2021-02-16

**Authors:** Jordam William Pereira-Silva, Claudia María Ríos-Velásquez, Gervilane Ribeiro de Lima, Eric Fabrício Marialva dos Santos, Heliana Christy Matos Belchior, Sergio Luiz Bessa Luz, Felipe Gomes Naveca, Felipe Arley Costa Pessoa

**Affiliations:** 1 Laboratório Ecologia de Doenças Transmissíveis na Amazônia, Instituto Leônidas e Maria Deane—Fiocruz Amazônia, Manaus, Amazonas, Brasil; 2 Programa de Pós-Graduação em Condições de Vida e Situações de Saúde na Amazônia, Instituto Leônidas e Maria Deane—Fiocruz Amazônia, Manaus, Amazonas, Brasil; 3 Fundação de Medicina Tropical Dr. Heitor Vieira Dourado, Manaus, Amazonas, Brasil; 4 Programa de Pós-Graduação em Medicina Tropical, Universidade do Estado do Amazonas, Manaus, Amazonas, Brasil; 5 Programa de Pós-Graduação em Biologia da Interação Patógeno-Hospedeiro, Instituto Leônidas e Maria Deane—Fiocruz Amazônia, Manaus, Amazonas, Brasil; Instituto Rene Rachou, BRAZIL

## Abstract

Mosquito diversity and disease transmission are influenced by landscape modifications, i.e., vectors and pathogens previously found only in forests are now found close to human environments due to anthropic changes. This study determined the diversity and distribution of mosquitoes in forest environments in order to analyze the potential vectors of Amazonian forest arboviruses. Mosquitoes were collected by 1) vertical stratification from forest canopy and ground areas using Hooper Pugedo (HP) light traps and human attraction and 2) horizontal stratification using HP light traps in peridomicile, forest edge, and forest environments near the Rio Pardo rural settlement, Amazonas, Brazil. A total of 3,750 mosquitoes were collected, representing 46 species. 3,139 individuals representing 46 species were sampled by vertical stratification. Both the Shannon-Weaver diversity index (H’) and equitability (J’) were higher in the canopy than on the ground. 611 individuals representing 13 species were sampled by horizontal stratification. H’ decreased in the following order: forest edge > forest > peridomicile, and J’ was greater at the forest edge and smaller in the peridomicile environment. Moreover, H’ was higher for the human attraction collection method than the HP traps. A total of 671 pools were analyzed by RT-qPCR; three species were positive for Oropouche-like viruses (*Ochlerotatus serratus*, *Psorophora cingulata*, and *Haemagogus tropicalis*) and the minimum infection rate was 0.8%. The composition of mosquito species did not differ significantly between anthropic and forest environments in Rio Pardo. Some mosquito species, due to their abundance, dispersion in the three environments, and record of natural infection, were hypothesized to participate in the arbovirus transmission cycle in this Amazonian rural settlement.

## Introduction

Mosquitoes are medically and veterinary important insects because they transmit arboviruses, protozoa, helminths, and other pathogens that impact public health [1]. In the Amazon region, mosquito species are diverse and the relationship between mosquito diversity and disease transmission is influenced by landscape changes related to deforestation, road construction, establishment of settlements, diversification of production activities related to family farming, and unplanned dwellings [2].

Environmental modifications can trigger two phenomena related to arbovirus survival and maintenance: (1) they can cause arboviruses to spread and infect humans and, depending on the distribution and availability of competent vectors, lead to epidemics, and (2) disappearance of arboviruses due to changes in the life cycle development of mosquitoes, affected by changes in some abiotic variables, such as temperature and because deforestation may cause the disappearance of wild animals [3]. Furthermore, the emergence and resurgence of arboviruses are often not only related to environmental changes, such as deforestation, but also to land use and the geographical location of households within a community area [2, 4]. Replacing forests with crops, livestock, and small animal breeding can create suitable habitats for mosquito proliferation and increase the risk for pathogen transmission to humans [5].

The definition of risk areas for infected vector exposure has increased considerably [6] because mosquito distribution is affected by environmental characteristics that favor changes in climate and landscape [4]. These modifications might induce favorable conditions for disease spread (e.g. increase human-mosquito contact rates, increased environmental suitability for the mosquito vector and reservoir hosts) [7]. Therefore, knowledge of mosquito fauna as well as evidence of natural infection are important for the design of vector control strategies to avoid or control disease outbreaks.

The construction of rural settlements in Brazil is a major cause of deforestation; the number of such settlements increased by 70% between 2002 and 2014, from 220.000 km^2^ to 376.000 km^2^ [8]. The Rio Pardo community is a typical rural settlement of the Brazilian Amazon that has experienced economic and demographic changes through intense deforestation as a result of opening new roads, fish farming dams, and agriculture and livestock farming. These factors can lead to outbreaks of arboviruses. A study conducted in the Rio Pardo community demonstrated that 44% of local inhabitants were seropositive for arboviruses such as Mayaro virus (MAYV), including children less than five years old [9]. Thus, the authors hypothesized that mosquitoes associated with the transmission of MAYV may circulate in anthropic or peridomiciliary environments.

Despite great concern about infections caused by Dengue virus (DENV), Zika virus (ZIKV) and Chikungunya virus (CHIKV), reports show that the MAYV and Oropouche virus (OROV) deserve special attention, especially in the northern region of South America [9–12]. Before the ZIKV and CHIKV epidemics in Brazil, Oropouche fever was considered the second arbovirus infection with the most reports in the country [13, 14]. Although there is no evidence of human infected by OROV in the Rio Pardo rural settlement in the Amazon region many people have antibodies which may indicate a large circulation of this arbovirus [15]. Furthermore, it is believed that most of the non-conclusive cases for DENV, ZIKV and CHIKV are caused by OROV [16, 17].

The OROV belongs to the *Peribunyaviridae* family, genus *Orthobunyavirus* [18]. It has a wild cycle involving wild vertebrate animals such as birds, monkeys, sloths and rodents and invertebrate hosts such as *Ochlerotatus serratus* and *Coquillettidia venezuelensis*. In the urban cycle, humans can act as amplifying hosts and *Culicoides paraensis* as the main vector. In addition, *Culex quinquefasciatus* appears to be an urban vector for OROV [11, 19].

The objectives of this work are: (1) to evaluate the diversity and distribution patterns of mosquitoes by vertical stratification in forest environments and horizontal stratification in peridomicile, forest edge and forest; (2) to identify species of MAYV, OROV, and other OROV-like viruses carrying the OROV S segment in a rural Amazon settlement in Brazil.

## Materials and methods

### Study site

**Fig 1:**
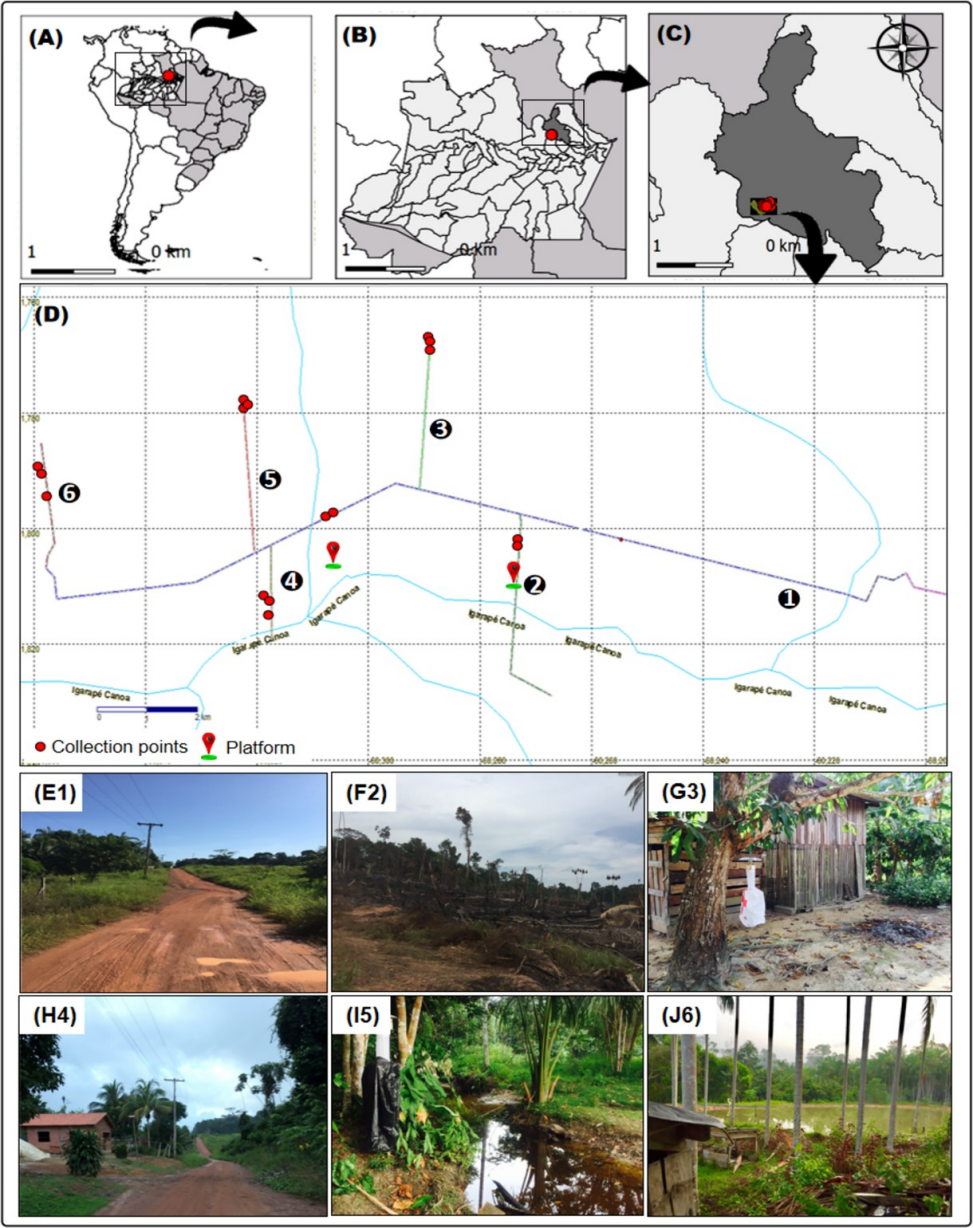
Map of mosquito collection area in Rio Pardo, Presidente Figueiredo, Amazonas State, Brazil. (A) South America; (B) Amazonas State; (C) Presidente Figueiredo Municipality; (D) Study area: The image shows the six branches of the community. The red dots represent the collection sites by horizontal stratification and the red and green symbols represent the platforms in the forest for collections by vertical stratification; (E1) Roads: Principal; (F2) Gusmão; (G3) Terra preta; (H4) Samuel; (I5) Novo Paraíso; (J6) Taxista.

The Rio Pardo rural settlement is in Presidente Figueiredo Municipality, Amazonas State, Brazil. The settlement was created in a dense area of Terra-Firme (non-flooded) Rainforest with a total area of 28,000 hectares. The population was 584 in September, 2019, and the area comprises six roads: Principal (1°48’03.1"S, 60°15’30.7"W) (Fig 1E1), Maria Gusmão (1°49’11.3"S, 60°15’26.6"W) (Fig 1F2), Terra Preta (1°46’46.7"S, 60°17’23.7"W) (Fig 1G3), Samuel (1°49’02.3"S, 60°19’03.5"W) (Fig 1H4), Novo Paraíso (1°47’24.9"S, 60°19’16.4"W) (Fig 1I5), and Taxista (1°47’57.8"S, 60°21’20.4"W) (Fig 1J6). The main economic activities are agriculture, resource extraction, and livestock rearing [20]. The rate of forest clearing in the legal Amazon between 2000 and 2013 was estimated as 180,332,66 hectares [21]. The climate is classified as Af according to the Köppen classification scheme, with a mean annual temperature of 27°C.

### Collection of mosquitoes

Due to logistical convenience, the collection efforts were done during four months, June and July, dry season, and November and December, rainy season, of 2016. Vertical and horizontal stratification transects were used to collect the mosquitoes (S1 Fig). The collection of mosquitoes was authorized by the Biodiversity Authorization and Information System (SISBIO), register: 12186.

### Vertical stratification method of mosquito collection

The two platforms were built in the canopy of trees 12m above ground level in an area of primary forest. The places for platforms installation were due to the similarities between the local trees and canopy cover.

The mosquitoes were collected from the ground and canopy levels using two methods: 1) by Hooper Pugedo (HP) light traps, installed overnight, from 18:00–06:00 over four consecutive nights per sampled month [22], and 2) by the standard human landing catch (HLC) method in the daytime from 06:00–08:00 and 14:00–16:00 and at night from 17:00–19:00. Canopy collections were conducted using two platforms built in forested areas at a height of approximately 12 m, one in Gusmão (1°49’11.3"S, 60°15’26.6"W) (Fig 1F2) and one in Principal (1°48’03.1"S, 60°15’30.7"W) (Fig 1E1) over five consecutive days (Fig 1).

In the text we use the abbreviations: HP-ground and HP-canopy to indicate the different HP light traps and HLC-ground and HLC-canopy to indicate the different HLC methods.

### Horizontal stratification method of mosquito collection

Mosquito collection along horizontal stratification transects was performed using HP light traps installed 1.5 m off the ground from 18:00–06:00 over four consecutive nights per sampled month in three different environments: peridomicile, forest edge, and forest. The three environments were defined as follows: peridomicile = external area of a residence, within a radius not exceeding 100 m [23]; forest edge = region of contact between the occupied area (anthropic matrix) and the fragment of natural vegetation [24, 25]; and forest = area of closed forest within preserved forest.

After each collection event, mosquitoes were anesthetized at cold temperatures in the field laboratory for identification at the species level using dichotomous keys [26–28], then conserved in 1 mL of RNAlater^™^ before being taken to a laboratory.

### Detection of Oropouche-like viruses in mosquitoes

The collected females were grouped in pools of up to 10 mosquitoes by identified species. The pools were processed for RNA extraction using 1 mL of TRIzol^®^ Reagent, and the RNA was extracted according to the manufacturer’s instructions. The viral RNA was detected using TaqMan^®^ Fast Virus 1-Step Master Mix in a StepOnePlus Real-Time PCR System (Applied Biosystems) using MAYV and OROV primers and probes [29]. All mosquitoes were subjected to a sensitive and specific method (RT-qPCR) for the detection of MAYV, OROV, and other OROV-like viruses carrying the OROV S segment [9, 10, 29, 30].

The RT-qPCR conditions were 50°C for 5 min, 95°C for 20 s, then 45 cycles of 95°C for 3 s, and 60°C for 30 s with fluorescence acquisition. For all RT-qPCR assays, the MS2 RNA bacteriophage was spiked prior to RNA extraction to track false-negative reactions due to PCR inhibition, as described elsewhere [29]. The RT-qPCR reactions were analyzed based on the cycle threshold (CT) values described by Naveca et al. [29], who considered a Ct value lower than 38 as positive (mean Ct values, 34.9–38.3 and 34.8–38.1 for MAYV and OROV, respectively). Positive MAYV and OROV samples were used for positive control. Data were analyzed using the QuantStudio 5 Real-Time PCR System.

### Statistical analysis

To evaluate the diversity species the Shannon-Weaver (H’) was estimated. This index is used in situations where the community total cannot be inventoried. The higher the H’ value, the greater the species diversity. This index can express wealth and uniformity [31]. Additionally, the Rényi diversity index was used to compare species diversity and evenness between environments (ground and canopy), and the Mann-Whitney test was used to compare species diversity in the ground and canopy of the platforms. The Pielou Equitability Index (J’) was used to evaluate the distribution of individuals among existing species.

To verify the influence of the environment (peridomicile, forest edge and forest) on the species composition of mosquitoes, Permutational Multivariate Analysis of Variance (PERMANOVA) was employed. A Non-Metric Dimensional Scaling (NMDS) method with the Bray-Curtis index was used to represent the position of these communities in a multidimensional space [32].

The rarefaction curve was used to calculate the sampling effort as a function of the frequency of captured individuals to determine whether the number of mosquitoes collected reached the point where the species richness is saturated [33].

The minimum infection rate in mosquitoes was obtained with PooledInfRate, version 4.0, using both the Maximum Likelihood Estimation (MLE) and Minimum Infection Rate (MIR) methods (https://www.cdc.gov/westnile/resourcepages/mosqsurvsoft.html).

Statistical analyses were carried out using the program free statistical software Version 1.2.1335 with the vegan, labdsv, ggplot2 and Past Version 3.14 [34–38].

## Results

At all 3,750 mosquitoes distributed in two subfamilies, Anophelinae and Culicinae, were collected. The subfamilies collected, Culicinae was the one with the highest number of individuals (94.46%), while Anophelinae represented only 5.54%.

The mosquitoes were distributed among six tribes (Aedeomyiini, Aedini, Sabethini, Culicini, Uranotaeniini, and Mansoniini), 17 genera (*Aedes*, *Aedeomyia*, *Anopheles*, *Coquillettidia*, *Culex*, *Haemagogus*, *Johnbelkinia*, *Limatus*, *Mansonia*, *Ochlerotatus*, *Orthopodomyia*, *Psorophora*, *Runchomyia*, *Sabethes*, *Trichoprosopon*, *Uranotaenia*, and *Wyeomyia*), and 46 species (Tables 1 and 2). The number of species may be higher; however, it was not possible to identify *Culex* of the subgenus *Melanoconion* due to difficulties and taxonomic limitations.

**Table 1:** Number of mosquitoes collected from the ground and canopy in platforms located at Principal and Gusmão roads in the rural settlement of Rio Pardo, Presidente Figueiredo, Amazonas State, Brazil, in 2016.

Species	Collections				
	HLC-ground	HLC-canopy	HP Ground	HP Canopy	Total
*Aedes (Stg*.*) albopictus*	2	1	0	0	3
*Aedes sp*	5	0	0	0	5
*Anopheles (Nys*.*) oswaldoi*	0	0	2	0	2
*Anopheles (Ano*.*) eiseni*	1	0	0	0	1
*Anopheles (Ano*.*) mattogrossensis*	1	0	0	7	8
*Anopheles (Ano*.*) mediopunctatus*	16	49	7	3	75
*Anopheles (Ste*.*) nimbus*	73	1	2	1	77
*Anopheles (Nys*.*) nuneztovari*	3	0	0	0	3
*Anopheles (Ano*.*) peryassui*	0	1	0	0	1
*Anopheles (Nys*.*) rondoni*	0	1	0	0	1
*Anopheles (Lop*.*) squamifemur*	0	1	0	0	1
*Anopheles (Nys*.*) triannulatus*	19	8	5	7	39
*Coquillettidia (Rhy*.*) venezuelensis*	3	0	2	0	5
*Coquillettidia (Rhy*.*) arribalzagae*	48	0	1	0	49
*Coquillettidia (Rhy*.*) lynchi*	35	0	1	0	36
*Coquillettidia (Rhy*.*) nigricans*	0	0	1	0	1
*Culex (Melanoconion) sp*	90	110	47	50	297
*Culex (Cux*.*) coronator*	0	0	2	0	2
*Culex (Cux*.*) nigripalpus*	12	2	17	15	46
*Haemagogus sp*	0	7	0	0	7
*Haemagogus (Hag*.*) janthinomys*	8	35	0	0	43
*Haemagogus (Hag*.*) tropicalis*	1	27	0	0	28
*Johnbelkinia longipes*	65	7	0	1	73
*Limatus durhamii*	8	0	0	2	10
*Limatus pseudomesthysticus*	70	1	0	0	71
*Mansonia (Man*.*) titillans*	1	0	0	2	3
*Ochlerotatus (Och*.*) fulvus*	54	9	2	4	69
*Ochlerotatus (Och*.*) argyrothorax*	7	1	0	0	8
*Ochlerotatus (Och*.*) fulvithorax*	82	0	3	2	87
*Ochlerotatus (Och*.*) scapularis*	4	0	0	0	4
*Ochlerotatus (Och*.*) serratus*	503	20	34	19	576
*Orthopodomyia fascipes*	1	1	4	1	7
*Psorophora (Jan*.*) albipes*	84	103	1	0	188
*Psorophora (Pso*.*) ciliata*	0	1	0	0	1
*Psorophora (Pso*.*) cilipes*	8	4	3	0	15
*Psorophora (Gra*.*) cingulata*	225	3	24	6	258
*Psorophora (Gra*.*) dimidiata*	14	0	0	0	14
*Psorophora (Jan*.*) ferox*	665	70	5	0	740
*Psorophora (Pso*.*) saeva*	2	0	1	0	3
*Runchomyia sp*	1	0	0	0	1
*Sabethes (Sbo*.*) chloropterus*	0	20	0	0	20
*Sabethes (Sbo*.*) glaucodaemon*	0	3	0	0	3
*Sabethes (Sab*.*) albiprivus*	2	32	0	0	34
*Sabethes (Sab*.*) amazonicus*	0	4	0	0	4
*Sabethes (Sab*.*) belisarioi*	0	9	0	0	9
*Sabethes sp*	0	13	0	0	13
*Trichoprosopon (Trc*.*) digitatum*	7	1	0	0	8
*Trichoprosopon sp*	2	2	0	0	4
*Uranotaenia (Ura*.*) calosomata*	0	1	0	0	1
*Uranotaenia (Ura*.*) geometrica*	0	0	1	0	1
*Wyeomyia (Den*.*) aporonoma*	18	1	0	0	19
*Wyeomyia sp*	149	12	4	0	165
Abundance	2289	561	169	120	3139
Richess	31	28	19	12	
Diversity (H’)	2.17	2.42	2.21	2.09	
Equitability (J’)	0.63	0.72	0.75	0.84	

HP: Hooper Pugedo, HLC: Human landing catch.

**Table 2:** Number of mosquitoes collected in the horizontal stratification transect by HP traps on six roads in the rural settlement of Rio Pardo, Presidente Figueiredo, Amazonas, Brazil, in 2016.

Species					
		Environment			
	Peridomicile	Forest edge	Forest	Total	%
*Aedeomyia squamipennis*	2	1	0	3	0.49
*Anopheles (Nys*.*) oswaldoi*	0	1	0	1	0.16
*Coquillettidia (Rhy*.*) venezuelensis*	0	3	30	33	5.40
*Coquillettidia (Rhy*.*) nigricans*	0	0	1	1	0.16
*Culex (Melanoconion)* sp	142	54	86	282	46.15
*Culex (Cux*.*) nigripalpus*	7	16	28	51	8.35
*Culex (Cux*.*) quinquefasciatus*	149	29	6	184	30.11
*Ochlerotatus (Och*.*) fulvithorax*	2	1	1	4	0.65
*Ochlerotatus (Och*.*) scapularis*	0	2	0	2	0.33
*Ochlerotatus (Och*.*) serratus*	0	9	5	14	2.29
*Psorophora (Jan*.*) albipes*	1	4	1	6	0.98
*Psorophora (Gra*.*) cingulata*	1	4	0	5	0.82
*Uranotaenia (Ura*.*) calosomata*	0	1	0	1	0.16
*Uranotaenia (Ura*.*) geometrica*	1	2	19	22	3.60
*Wyeomyia* sp	0	0	2	2	0.33
Abundance	305	127	179	611	100
Richess	7	12	8		
Diversity (H’)	0.91	1.73	1.53		
Equitability (J’)	0.43	0.67	0.66		

The most abundant genera were *Psorophora*, with seven species and 1,230 individuals (32.8%), *Culex*, with four species and 899 individuals (23.9%), and *Ochlerotatus*, with five species and 744 individuals (19.8%). The least represented genus was *Runchomyia* and *Mansonia* with one individual each. *Psorophora ferox* showed the highest abundance with 740 individuals (19.7%), followed by *Oc*. *serratus* with 590 (15.7%), and *Ps*. *cingulata* with 263 (7%) The number of *Culex* females of the subgenus *Melanoconion* was 606 (16.1%).

### Vertical stratification

A total of 3,139 specimens belonging to 46 species were collected from platforms of Gusmão and Principal roads located on the ground and canopy (Table 1). In general, the abundance and richness were highest on the ground (Table 1). The species richness varied from 12 to 31 among the ground and canopy, and the abundance varied from 120 to 2,289 individuals (Table 1). The most abundant genera were *Psorophora*, with seven species and 1,219 specimens (31.8%), and *Ochlerotatus* with five species and 744 specimens (19.8%). The most abundant species were *Ps*. *ferox*, with 740 individuals (23.5%), *Oc*. *serratus*, with 576 (18.3%), and *Ps*. *cingulata* with 258 (8.2%); these were the most abundant at all strata (ground and canopy), demonstrating that these species travel between different elevations.

**Fig 2:**
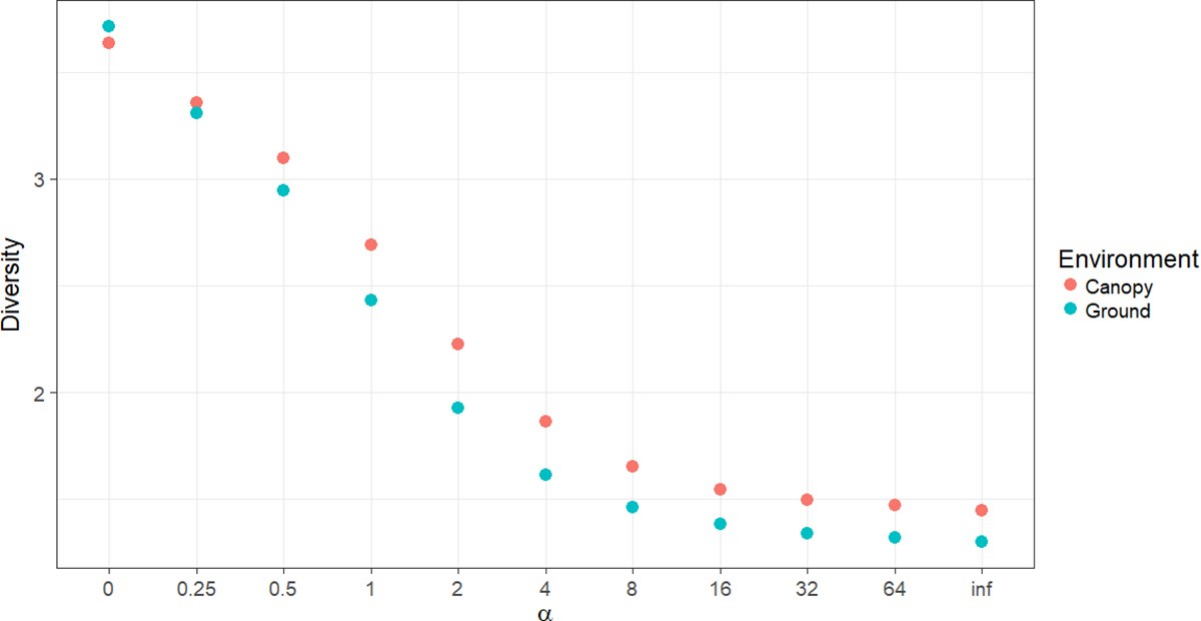
Rényi diversity profiles on the ground and canopy in Rio Pardo, Presidente Figueiredo, Amazonas State, Brazil. α-values: zero = log wealth, 1 = Shannon Index.

Independent of the collection method, the species diversity was highest in the canopy (Fig 2). More details of the diversity index (H’) for each environment and the methods of collection are shown in Table 1. The equitability index (J’) for HP-ground and HP-canopy was greater than that for HLC-ground and HLC-canopy (Table 1).

The HLC-ground collections involved 2,289 individuals belonging to 31 species. *Ps*. *ferox* showed the highest abundance with 665 individuals (29.0%), followed by *Oc*. *serratus* with 503 (21.9%). Conversely, HLC-canopy collections involved 561 individuals belonging to 28 species. The species *Ps*. *albipes* showed the highest abundance with 103 individuals (18.3%), followed by *Ps*. *ferox* with 70 (12.4%). A higher abundance was observed for HLC-ground; however, the diversity was higher in HLC-canopy. These differences were statistically significant (p = 0.037). The HP-ground collections involved 169 individuals belonging to 19 species. *Ochlerotatus serratus* showed the highest abundance with 34 individuals (20.2%), followed by *Ps*. *cingulata* with 24 (14.2%). HP-canopy collections involved 120 individuals belonging to 12 species. *Ochlerotatus serratus* again showed the highest abundance with 19 individuals (15.8%), followed by *Cx*. *nigripalpus* with 15 (12.5%). However, there were no statistically significant differences between the two environments (p = 0.059).

**Fig 3:**
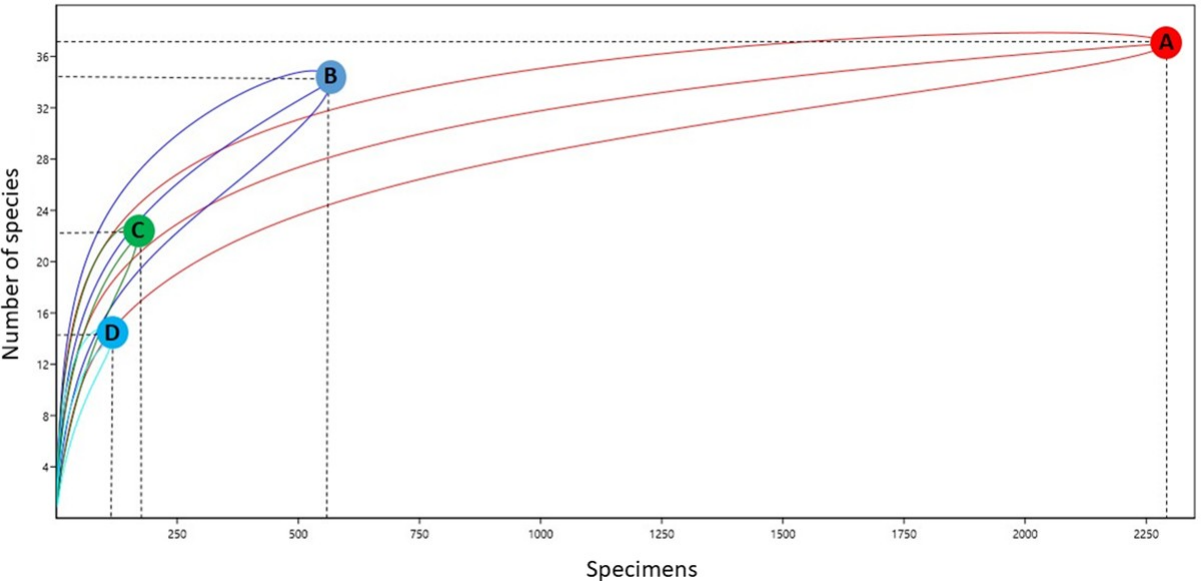
Rarefaction curves representing species richness of mosquitoes collected by vertical stratification. (A) HLC-ground; (B) HLC-canopy; (C) HP-ground; (D) HP-canopy.

Some specificities were observed in relation to the presence of different species on the ground and canopy. *Ochlerotatus serratus* was collected at both heights and with all collection methods. Other species such as *Cx*. *nigripalpus*, *Oc*. *fulvus*, and *Ps*. *cingulata* were also collected from both ground and canopy environments. In contrast, some species were found in only one environment, such as *Cq*. *venezuelensis*, *Cq*. *arribalzagae*, and *Cq*. *lynchi*, which were collected only on the ground, even when using different traps. The species captured by HLC-ground and HLC-canopy methods presented greater similarity (47%) than those captured by HP-ground and HP-canopy methods (38%). Species richness differed among the different environments and collection methods. According to the rarefaction curve, different levels of richness were observed between points with 95% CI (Fig 3). The rarefaction curve demonstrates that the richness of species collected by HP-ground, HP-canopy, and HLC-canopy methods may be greater than that of species collected by the HLC-ground method (Fig 3).

### Horizontal stratification

Along the horizontal stratification transect, 611 mosquitoes were collected, belonging to 13 species (Table 2). The richness of species varied from seven to 13 among the environments, and the abundance varied from 127 to 305 individuals (Table 2). The most abundant genera were *Culex*, with two species and 544 individuals (89.03%), *Uranotaenia*, with two species and 22 individuals (3.60%), and *Aedes* with three species and 20 individuals (3.27%). *Culex quinquefasciatus* showed the highest abundance with 184 individuals (30.11%), followed by *Cx*. *nigripalpus* with 51 (8.34%) and *Ur*. *colosomata* with 22 (3.60%).

In the peridomicile area, 305 specimens were collected, belonging to seven species. The most abundant species was *Cx*. *quinquefasciatus* with 149 individuals (48.85%), followed by *Cx*. *(Mel*.*)* sp with 142 (46.55%) and *Cx*. *nigripalpus* with 7 (2.3%). In the forest edge area, 127 specimens were collected, belonging to 12 species. The most abundant species was *Cx*. *quinquefasciatus* with 29 individuals (22.8%), followed by *Cx*. *nigripalpus* with 16 (12.60%) and *Oc*. *serratus* with 9 (7.09%). In the forest, 179 specimens were collected, belonging to eight species. The most abundant species was *Cx*. *nigripalpus* with 28 individuals (15.64%), followed by *Ur*. *geometrica* with 19 (10.61%) and *Oc*. *serratus* with 5 (2.79%). Some species were present in all environments, e.g. *Cx*. *quinquefasciatus*, *Cx*. *nigripalpus*, *Ps*. *albipes*, and *Oc*. *fulvithorax*. *Cq*. *venezuelensi*s and *Oc*. *serratus* were the only species found exclusively between the forest and forest edge. Furthermore, *Oc*. *scapularis*, *An*. *oswaldo*i, and *Ur*. *colosomata* were found exclusively at the forest edge. *Coquillettidia nigricans* was only found in the forest environment. The peridomicile environment did not exhibit any exclusive species (Table 2).

**Fig 4:**
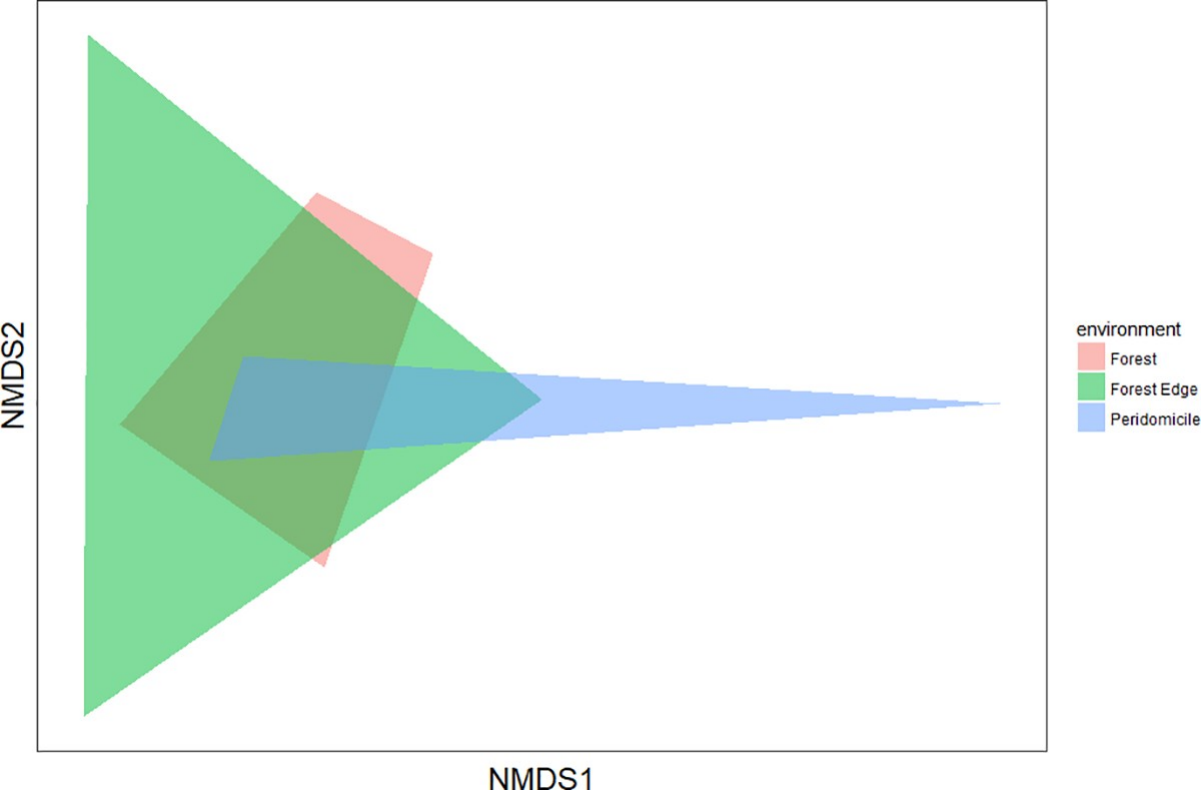
Non-Metric Multidimensional Scaling (NMDS) for the species collected in each environment of the Rio Pardo settlement, Presidente Figueiredo Municipality, Amazonas State, Brazil.

**Fig 5:**
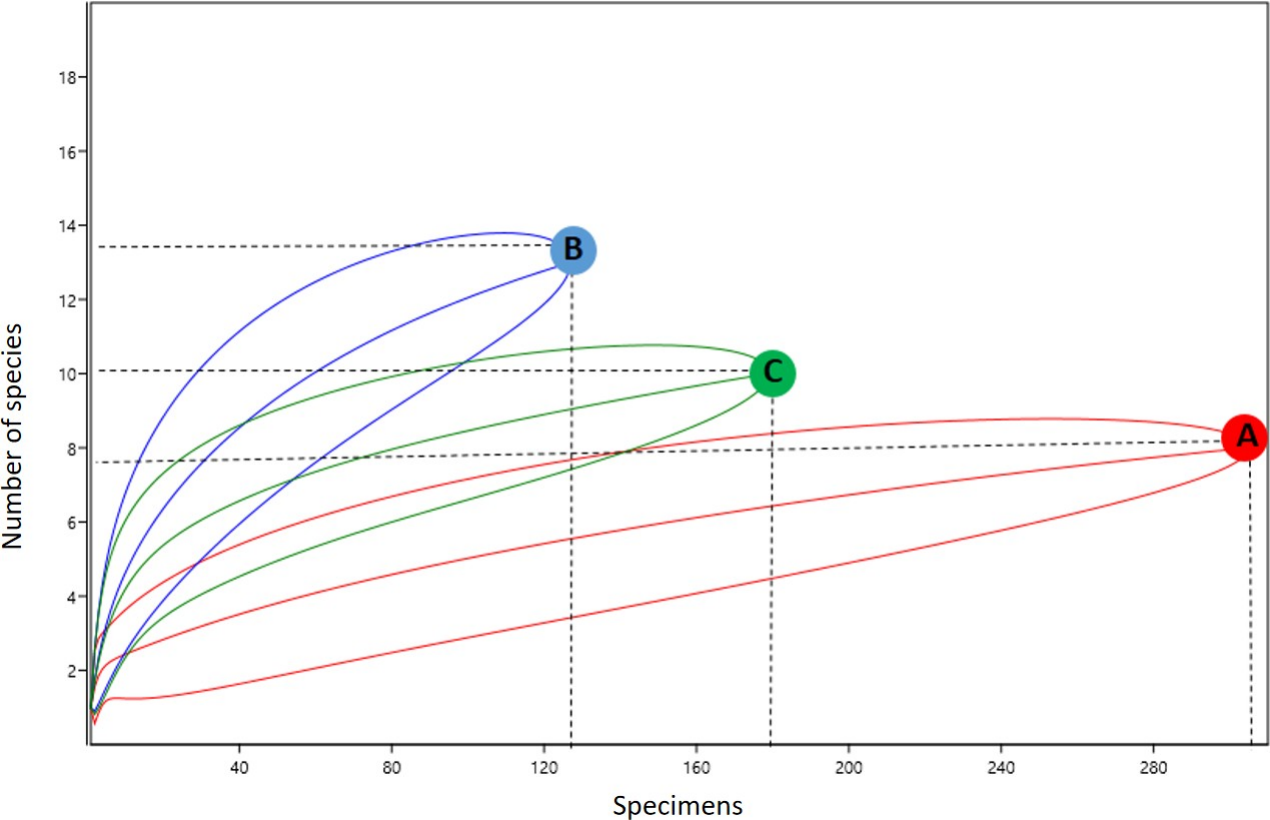
Rarefaction curves representing species richness of mosquitoes collected by horizontal stratification. (A) Peridomicile; (B) Forest edge; (C) Forest environment of the Rio Pardo settlement, Presidente Figueiredo Municipality, Amazonas State, Brazil.

Species diversity was highest at the forest edge (H’ = 1.73), followed by forest (H’ = 1.53) and peridomicile (H’ = 0.91) environments. The equitability index (J’) for forest edge and forest environments was greater than that in the peridomicile environment (Table 2), indicating that individuals were distributed among different species in the three environments. The forest and forest edge environments presented greater similarity of species (57% similarity), whereas peridomicile and forest showed smaller similarity (42% similarity). The NMDS revealed no significant difference among the three environments (PERMANOVA S.S = 0.69, pseudo-F = 1.42, p = 0.15) (Fig 4). The rarefaction curves showed that species richness in peridomicile, forest edge, and forest environments did not reach a plateau; therefore, the species richness can be greater depending on the sampling effort (Fig 5). The species richness also differed among the environments (95% CI) (Fig 5).

### Detection of Oropouche-like viruses in mosquitoes

In total, 3,750 females collected from platforms located on Gusmão and Principal roads and using HP light traps in peridomicile, forest edge, and forest environments were grouped in 671 pools of up to 10 mosquitoes. 532 pools (79.28%) were collected by vertical stratification and 139 pools (20.72%) were collected using HP light traps in the three different environments. The highest number of pools belonged to the species *Oc*. *serratus* (81 pools), *Ps*. *ferox* (80), and *Ps*. *cingulata* (41).

RT-qPCR showed that three of the 671 pools of mosquitoes were positive for viruses carrying the OROV S segment and no sample was positive for MAYV. The Minimum Infection Rate (MIR) was 0.8% per 1,000 specimens. The OROV S segment was detected in pools of *Ps*. *cingulata* with 10 individuals captured by AH-ground (C_T_ = 35.32), *Hg*. *tropicalis* with three individuals captured by AH-canopy (C_T_ = 36.18), and *Oc*. *serratus* with 10 individuals also captured by AH-ground (C_T_ = 25.12); all of these samples were collected from Gusmão roads. Two nucleotide sequencing attempts were made with the three positive samples; however, due to the low viral load, we call it an Oropouche-like virus, because it was not possible to sequence the fragments and confirm the homology.

## Discussion

### Effects of vertical stratification on diversity of mosquitoes and implications for disease transmission

In the Rio Pardo rural settlement, the captured mosquitoes species correspond to 27% of the species recorded in Amazonas [39]. In other areas near Manaus, 31, 39, and 27 mosquitoes species were registered [40–42], demonstrating high diversity in Rio Pardo. Studies with mosquito species that inhabit the tree canopy are important because many disease-causing pathogens in humans are present in enzootic cycles, which involve vertebrate hosts such as monkeys and birds that preferentially inhabit the forest canopy [43]. Therefore, humans can invade the transmission cycle and be bitten by an infected vector.

According to the results of the vertical stratification method, although a strong preference for heights in large trees was expected, greater richness and abundance was observed on the ground. In addition, the results demonstrated that HLC collection was more efficient than HP trap collection. *Psorophora ferox* was the most abundant species, accounting for 23.5% of all collected species. This species was abundant in other studies in the forest of Brasília National Park [44] and in northern Amazonas [45]. *Ps*. *ferox* was an incriminated vector of the Rocio virus (ROCV) in a previous encephalitis outbreak in São Paulo and was isolated for the Brejeira virus (BRJV) in Pará State, northern Brazil [46, 47]. Furthermore, the importance of *Ps*. *ferox* is enhanced by its potential role in the transmission of Venezuelan Equine Encephalitis Virus (VEEV), MAYV, and Yellow Fever Virus (YFV) in the Amazon basin [48–50]. *Ochlerotatus serratus* was the second most abundant species, representing 18.3% of all collected species. Other studies have already registered the species [40–42, 51]; however, this the first record of *Oc*. *serratus* abundance in the Amazon region. *Oc*. *serratus* is naturally infected with OROV, an arbovirus typically found in rural areas of the Amazon [52].

The highest abundance of mosquitoes occurred at ground level, where people and other large animals circulate; however, the species diversity was highest in the canopy (Table 1). Furthermore, some species of mosquitoes preferred to inhabit the canopy, whereas other exhibited a random distribution (Table 1). Some species such as *Sa*. *albiprivus* and *Hg*. *janthinomys* were captured at ground level, as shown in other studies [44, 53], suggesting that some species of mosquito go to ground level to search for aquatic habitats to oviposit their eggs or blood food. Independent of the collection method, the abundance and richness were highest on the ground (Table 1). Therefore, the presence of *Sa*. *albiprivus* and *Hg*. *janthinomys*, vectors of MAYV and YFV, on the ground may increase the risk of disease transmission to the human population of settlements. In addition to *Sa*. *albiprivus* and *Hg*. *janthinomys*, the following other species incriminated or suspected as vectors were recorded by vertical stratification: *Cx*. *quinquefasciatus*, *Cx*. *nigripalpus*, *Ae*. *fulvus*, *Oc*. *scapularis*, *Cq*. *venezuelensis*, *Ps*. *albipes*, *Sa*. *chloropterus*, and *Hg*. *tropicalis*, which agrees with the findings of other research in the Brazilian Amazon [41, 45].

This study revealed that several vector species travel between the ground and canopy (Table 1). Likely as a result of blood availability, some species exercised eclecticism, as shown by the similarity index between AH-ground and AH-canopy methods and HP-ground and HP-canopy methods, which were 47% and 38%, respectively.

The presence of these vector species on the ground could indicate an effect of human action processes that may have reduced the number of trees, thereby affecting the habitat of potential food sources and canopy breeding sites, forcing insects to get down or adapt to ground environments [7, 50]. In this study, *Hg*. *janthinomys*, a vector of YFV and MAYV, was captured in both ground and canopy environments. The presence of species that live in the canopy of trees and that were found in the ground is a risk factor for the human population. A study in Brazil captured a high abundance of *Hg*. *janthinomys* at ground level and in open fields [50]. Furthermore, it has been demonstrated that *Anopheles balabacensis* is present in both ground and canopy environments in Malaysia and may be transmitting *Plasmodium simium* to monkeys and humans [54].

According to the rarefaction curves, different levels of richness were observed (Fig 3), with AH-ground the only one category to reach a plateau. As the majority of rarefaction curves were still increasing, the number of mosquito species in these environments is likely to be higher. Another study conducted in the Brazilian Amazon rainforest demonstrated the largest rarefaction curves in the canopy environment [40].

The capture of *Anopheles* mosquitoes by human landing catch (HLC) in the Amazon region is common, but in this work their abundance was low (Table 1). It is probably due to several factors: 1) HP trap method is not the most suitable to collect this genus of mosquitoes; 2) HLC seems to be the best method for *Anopheles* collection, however our collection times are not biting peaks of *Anopheles* mosquitoes, and 3) the platforms were built away from lakes and streams [55].

### Effects of horizontal stratification on diversity of mosquitoes and implications for disease transmission

It was expected that mosquito abundance and richness obtained by horizontal stratification would be highest in the forest; however, this was not observed. The greatest abundance was observed in the peridomicile environment, and the greatest diversity was observed at the forest edge. Changes in habitats can increase the number of niches and promote a greater diversity of mosquitoes in environments near dwellings [4, 56]. Species composition is constantly changing [57]; thus, inadequate land use and changes in the vector-host relationship are a risk factor that may favor the emergence of diseases when a vector is introduced to a new habitat or exposed to a new host [4].

There are differences between the peridomicile, forest edge and forest and this may have influenced the abundance and richness of mosquitoes. The presence of aquatic breeding and domestic animals in the peridomicile can be favorable for mosquito breeding. The greatest abundance of *Cx*. *quinquefasciatus* and *Cx*. *(Mel*.*)* sp. in the peridomicile environment may be associated with the higher blood feeding resource availability caused by the presence of domestic animals such as dogs, chickens, and pigs. Also, human action can lead to mosquito breeding sites increasing near houses. This same effect has been observed in sand flies in Rio Pardo, indicating that deforestation process may favoring peridomiciliation of vectors [58]. Thus, humans are likely to be exposed to the same risks to be infected by an arbovirus in the peridomicile and forest environments.

The NMDS showed similarity between the three studied environments (Fig 4). The similarity index varied between 42% and 57% and indicated mosquito adaptation process to the environments. Species typically associated with forest environments were collected near residences, which is evidence that mosquito species are circulating in all environments; this increases the vulnerability of populations to infectious diseases such as arboviruses and malaria. It is common in these areas to find wild animals, such as monkeys and birds, living in the same space as humans. Furthermore, thinking about domestic activities, people build their houses strategically close to streams, which allows the formation of mosquito breeding sites and, at the same time, provides a constant source of blood for mosquitoes. A study in Australia indicated that anthropic activities have a sizable influence on mosquito communities, with most vector species living in grasslands created by humans. This remarkable influence of humans on land use has potential implications for the transmission of pathogens [4].

*Culex quinquefasciatus* was dispersed among all three environments, but predominant occurred in the peridomicile. Barbosa et al. [41] also observed an abundance of *Cx*. *quinquefasciatus* in this environment in Amazonas. Garcia-Rejon et al. [59] demonstrated that *Cx*. *quinquefasicatus* feeds on a variety of hosts, such as chickens, dogs, cats, pigs, and humans. This dispersion in all environments can be a crucial factor in the transmission of arboviruses to humans. Furthermore, *Cx*. *quinquefasciatus* is considered a vector for OROV [11] and West Nile virus (WNV) [60].

Although MAYV-infected mosquitoes have not been collected, the possibility of the virus circulating in the community cannot be ignored. The possible peridomiciliation of MAYV vectors and the use of alternative vectors in the rural settlement of Rio Pardo has previously been suggested [9]. Interestingly, the natural infection of *Cx*. *quinquefasciatus* by MAYV was subsequently demonstrated in Mato Grosso, Brazil [12]. Thus, this species seems to play an important role and may be directly associated with infection by MAYV and other arboviruses in the community.

Although *Cx*. *(Mel*.*)* sp. was collected in abundance, they were identified down to the section level due to taxonomic difficulties. However, studies carried out along Amazonian rivers also recorded a high abundance of species in this subgenus [61, 62]. Several mosquitoes of the subgenus *Melanoconion* are vectors or have already been found naturally infected by arbovirus, such as *Cx*. (*Mel*.) *taeniopus*, which is an enzootic vector of VEEV, *Cx*. (*Mel*.) *spissipes* found naturally infected with Mucambo Virus (MUCV) and *Cx*. (*Mel*.) *portesi* collected naturally infected by MUCV, Bimiti virus (BIMV), Caraparu virus (CARV) and Oriboca virus (ORIV) [63, 64].

*Culex nigripalpus* was the second most abundant species, dispersed among all three environments, but predominantly occurred in the forest. The presence of this species in the three environments is concerning because this species is considered to be a vector of WNV and Saint Louis encephalitis virus (SLEV) [65, 66]. Other species incriminated or suspected as vectors were also recorded: *Cx*. *nigripalpus*, *Oc*. *scapularis*, *Oc*. *serratus*, *Cq*. *venezuelensis*, and *Ps*. *albipes*.

The rarefaction curve indicates that the species number in the peridomicile, forest edge and forest environments are higher than we are showing, suggesting that more sampling effort could increase the number of species collected. Confalonieri & Neto [67] used a similar methodology and demonstrated stabilization of the rarefaction curve in 34 species collected from forest areas in the State of Pará.

The medical importance of *Oc*. *serratus*, *Ps*. *cingulata* and *Hg*. *tropicalis* is recognized; however, to achieve a registry of natural infection by viruses carrying the OROV S segment, the need for epidemiological surveillance of OROV infection by health authorities should be reinforced. This study contributes to our knowledge of the distribution of mosquitoes in Amazonas State and provides the first record in the literature of *Oc*. *serratus*, *Ps*. *cingulata*, and *Hg*. *tropicalis* infected with viruses carrying the OROV S segment.

### Limitation of the study

Limitations of the study are: the period of time when field collections are realized, the small sample size that influences the possibility to found arbovirus infected mosquitoes (it is common for arboviruses to infect <1 mosquito in 1000 [68]), and the difficulty to identify *Culex* (*Melanoconion)* mosquitoes to specific level.

## Conclusion

In Rio Pardo, the species composition of mosquitoes between anthropic and forest environments does not exhibit a statistically significant difference. This highlights that vector mosquito species can participate in the transmission cycle of arboviruses in rural settlements. The abundance of *Oc*. *serratus* throughout all three environments and its record of natural infection by viruses carrying the OROV S segment indicate that this species is a possible vector for arboviruses in the Rio Pardo rural settlement, Amazonas, Brazil.

It is important that future studies determine the relationships between human activities, arboviruses, and their respective vectors in areas of rural settlement construction in order to consider the effect of direct contact between humans and mosquito vectors and reservoirs.

## Supporting information

S1 Fig (TIF)

S2 Fig (TIF)
