# Supplementary figures and images for: Distribution and diversity of mosquitoes and Oropouche-like virus infection rates in an Amazonian rural settlement
Source: PLoS One. 2021 Feb 16;16(2):e0246932. doi: 10.1371/journal.pone.0246932 (PMC7886159; doi:10.1371/journal.pone.0246932)

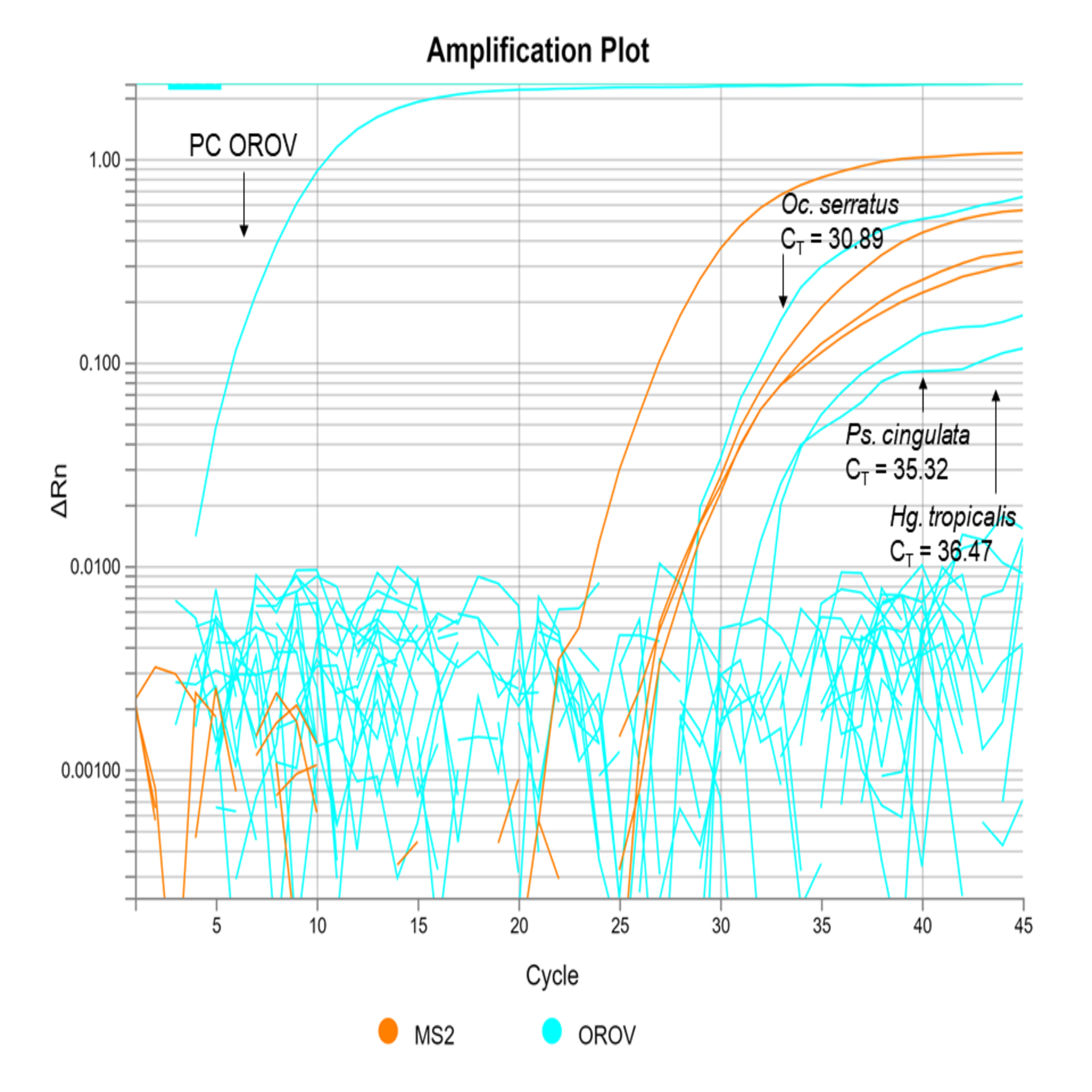

Supplement: S1 Fig — (TIF) [file pone.0246932.s001.tif]

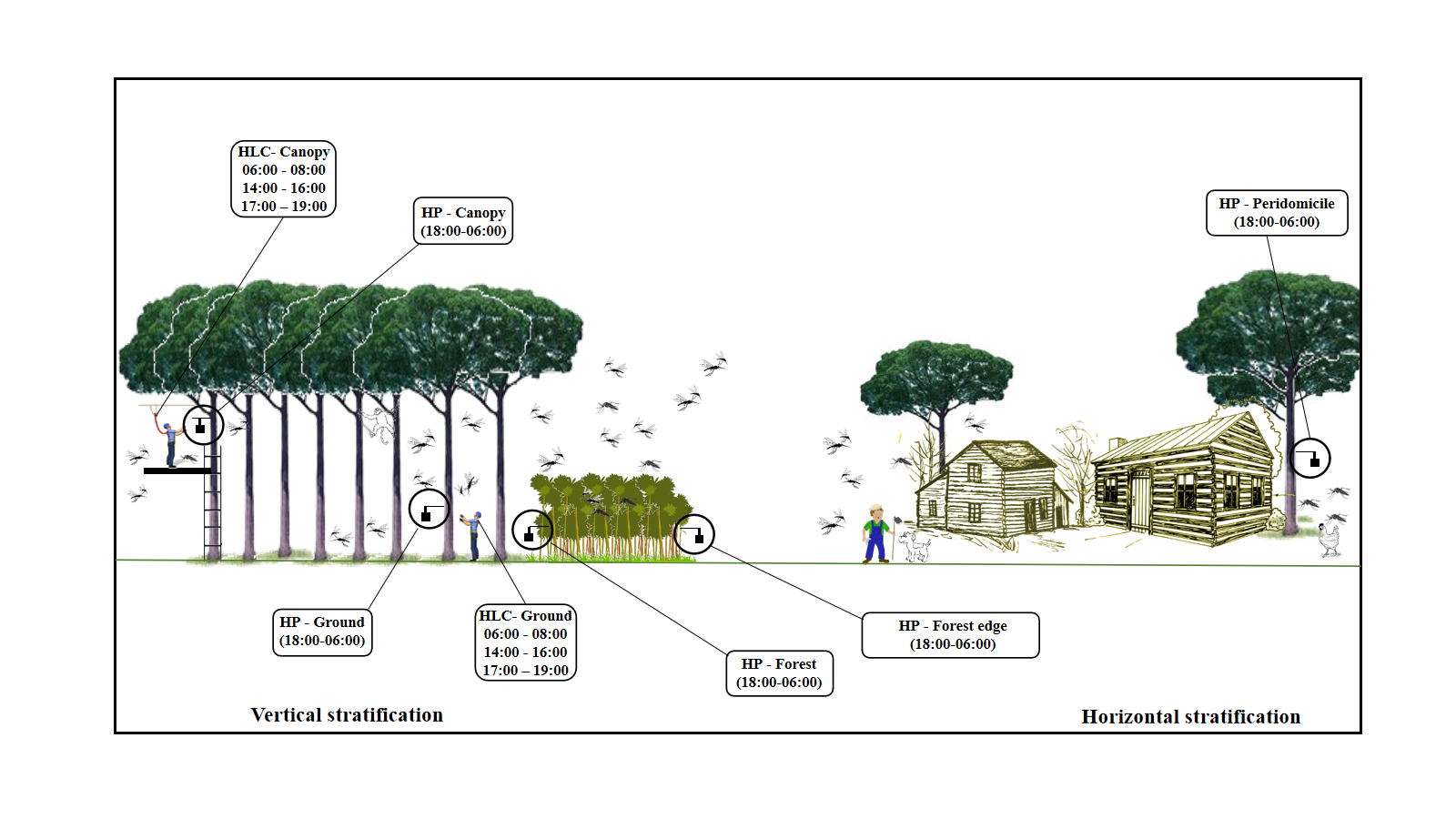

Supplement: S2 Fig — (TIF) [file pone.0246932.s002.tif]
